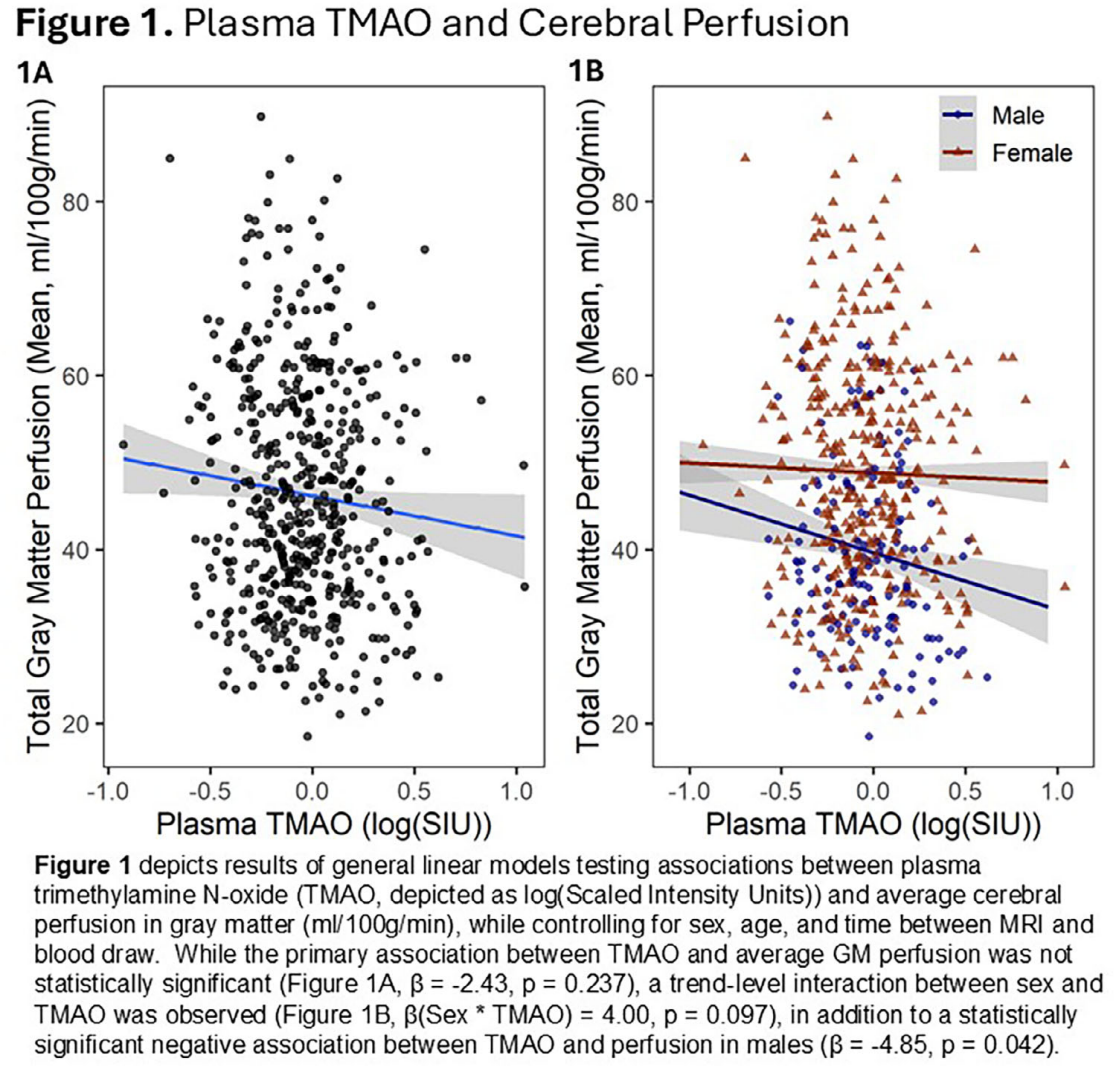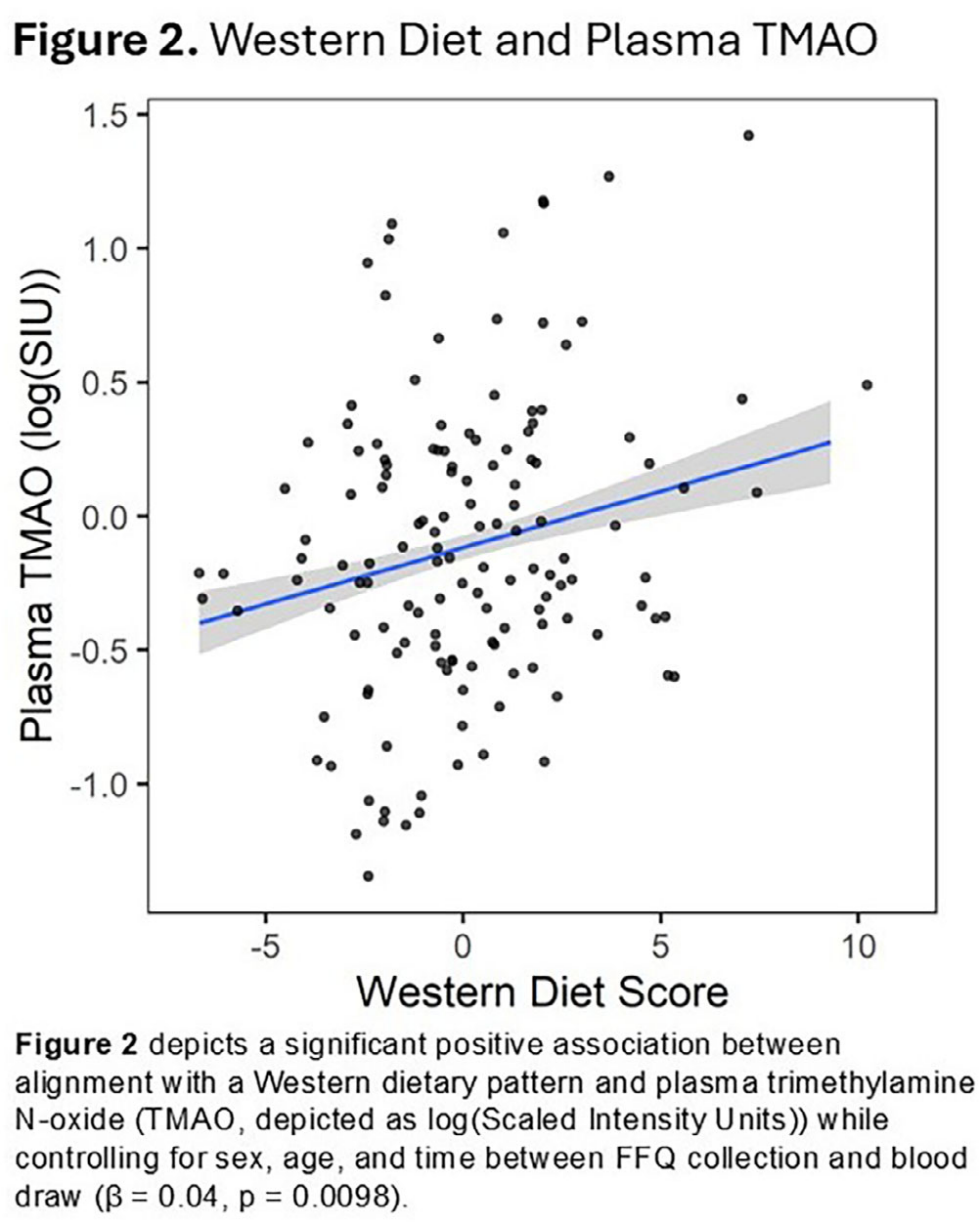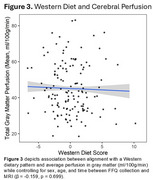# Plasma trimethylamine N‐oxide associates with Western dietary pattern and reduced cerebral perfusion in cognitively unimpaired adults

**DOI:** 10.1002/alz70861_108636

**Published:** 2025-12-23

**Authors:** Darby Peter, Briana L Rocha, Puja Agarwal, Leonardo A. Rivera‐Rivera, Kevin M. Johnson, Sanjay Asthana, Sterling C Johnson, Tyler K. Ulland, Corinne D. Engelman, Federico E. Rey, Barbara B. Bendlin

**Affiliations:** ^1^ Wisconsin Alzheimer's Disease Research Center, University of Wisconsin School of Medicine and Public Health, Madison, WI USA; ^2^ Neuroscience Training Program, University of Wisconsin‐Madison, School of Medicine and Public Health, Madison, WI USA; ^3^ University of Wisconsin‐Madison, Madison, WI USA; ^4^ Rush Alzheimer's Disease Center, Rush University Medical Center, Chicago, IL USA; ^5^ University of Wisconsin School of Medicine and Public Health, Madison, WI USA; ^6^ Wisconsin Alzheimer's Disease Research Center, University of Wisconsin‐Madison, School of Medicine and Public Health, Madison, WI USA; ^7^ Wisconsin School of Medicine and Public Health, Department of Pathology and Laboratory Medicine, Madison, WI USA; ^8^ Department of Bacteriology, University of Wisconsin‐Madison, Madison, WI USA

## Abstract

**Background:**

Recent evidence suggests that a Western dietary pattern associates with gut dysbiosis, increased amyloid pathology, and decreased cerebral perfusion in cognitively unimpaired adults. Additionally, altered gut microbial composition and increased levels of gut‐modulated trimethylamine N‐oxide (TMAO) are observed in individuals with Alzheimer’s disease and related dementias (ADRD). While TMAO levels are also increased in vascular conditions including atherosclerosis and stroke, the extent to which TMAO drives relationships between a Western diet and *in vivo* indicators of cerebrovascular health, including cerebral perfusion, needs further investigation. The goal of this study was to test associations between plasma TMAO and cerebral perfusion in gray matter, and test whether TMAO mediates the effect of a Western dietary pattern on cerebral perfusion in an aging cohort enriched for dementia risk.

**Method:**

Cognitively unimpaired participants from the Wisconsin Registry for Alzheimer’s Prevention and Wisconsin Alzheimer’s Disease Research Center (n = 513, mean ± SD age = 66.8 ± 7.8, 28% male) provided plasma samples and underwent magnetic resonance imaging (MRI), including pseudocontinuous arterial spin labeling (pcASL) MRI. A subset of participants (n = 141) also provided responses to a semiquantitative food frequency questionnaire (FFQ), from which Western diet scores were derived using factor analysis. Plasma TMAO was quantified via an untargeted metabolomics analysis (Metabolon, Inc., Durham, NC, USA) using ultrahigh performance liquid chromatography tandem mass spectrometry (UHPLC‐MS), with TMAO levels expressed as Scaled Intensity Units (SIU). Cerebral perfusion values were derived from multi‐delay pcASL and scaled to absolute cerebral blood flow (ml/100g/min) using a proton density (PD) reference scan.

**Result:**

We observed a significant negative association between plasma TMAO and average cerebral perfusion in gray matter in males (Figure 1), and a positive association between alignment with a Western dietary pattern and plasma TMAO (Figure 2). The association between Western diet and cerebral perfusion was not statistically significant (Figure 3).

**Conclusion:**

While no significant association was observed between Western diet scores and perfusion, Western diet was associated with higher TMAO, and higher TMAO was associated with lower perfusion among males. Future mechanistic studies are needed to determine if reducing TMAO beneficially impacts brain blood flow.